# Bridge enhanced ACL repair vs. ACL reconstruction for ACL tears: A systematic review and meta-analysis of comparative studies

**DOI:** 10.1051/sicotj/2023007

**Published:** 2023-04-13

**Authors:** Jad Mansour, Joe Ghanimeh, Ali Ghoul, Michel Estephan, Alfred Khoury, Mohammad Daher

**Affiliations:** 1 Division of Orthopaedic Surgery and Sports Medicine, McGill University Health Centre 845 Sherbrooke Street Montreal H4A 3J1 Quebec Canada; 2 Department of Orthopedic Surgery, Lebanese American University Medical Center‑Rizk Hospital, Lebanese American University School of Medicine Zahar Street 11-3288 Achrafieh Lebanon; 3 Hôtel Dieu de France, Orthopedic Department, Alfred Naccache Boulevard 166830 Beirut Lebanon

**Keywords:** Bridge enhanced ACL repair, ACL reconstruction, ACL repair, ACL tear, ACL graft

## Abstract

*Introduction*: Anterior cruciate ligament (ACL) tear is one of the most frequent ligamentous injuries. The gold standard for ACL tears is autograft reconstruction. However, ACL repair has regained enthusiasm with more recent results showing comparable outcomes to its reconstructive counterpart. *Methods*: PubMed, Cochrane, and Google Scholar (pp. 1–20) were searched until November 2022. The clinical outcomes consisted of the International Knee Documentation Committee (IKDC) score, Knee Injury and Osteoarthritis Outcome Score (KOOS), the side-to-side difference in Anteroposterior (AP) knee laxity, the forces of the hamstring, quadriceps, and hip abduction as well as hopping tests. *Results*: Only two studies were included in this meta-analysis. ACL repair was shown to have better Hamstrings strength. The rest of the analyzed outcomes were comparable. *Discussion*: This is the first meta-analysis comparing these two treatments. The ACL repair showed no differences in muscle strength (quadriceps and hip abductors), postoperative knee scores, and knee joint laxity when compared to ACL reconstruction. However, it showed better hamstring strength. Further randomized clinical studies will be needed to compare both of these techniques.

## Introduction

Anterior cruciate ligament (ACL) tear is one of the most frequent ligamentous injuries [1], with authors commonly reporting 100,000–200,000 cases per year in the United States, nearly half of which are treated surgically [1–3]. In this context, a plethora of surgical procedures has been described, from ACL repair, first described in 1903 by Mayo Robson [4], to ACL reconstruction (ACLR) using various grafts [5].

Following ACL rupture, the lack of mechanical intra-articular support of the torn ends, along with its hypovascular nature and the hostile synovial fluid environment compromise the primary healing of the ligament [6, 7]. This intrinsically poor healing potential led to a fall of interest in ACL repair favoring a gold standard ACL autograft reconstruction [6]. ACL repair has however regained enthusiasm with more recent results showing comparable outcomes to its reconstructive counterpart [8–10] with notable advantages such as avoiding donor-site morbidity while preserving the ACL native anatomical insertions and innervation.

Current ACL repair techniques include Suture anchor fixation [9] where the proximal end of the ligament is fixed back to its femoral insertion with anchor sutures; Tape reinforcement [11] in which a tape is used as a stabilizing internal ACL brace; and Dynamic Intraligamentary Stabilization [12] where a strong suture fixed to the tibia with spring screw system is used. In an attempt to optimize the biological environment for ACL healing following repair, a novel bridge-enhanced ACL repair (BEAR) had been developed [13]. It adjuncts a bioactive scaffold to suture repair, filling the gap between torn ends of the ligament.

The primary objective of our study is to undergo a systematic review and meta-analysis of relevant literature aiming to compare the functional outcomes, knee laxity, and muscle strength of the BEAR technique compared to the ACLR technique. The secondary aim of the study is to compare return to sport and psychological readiness postoperatively between those two techniques.

## Material and methods

### Search strategy

**Figure 1:**
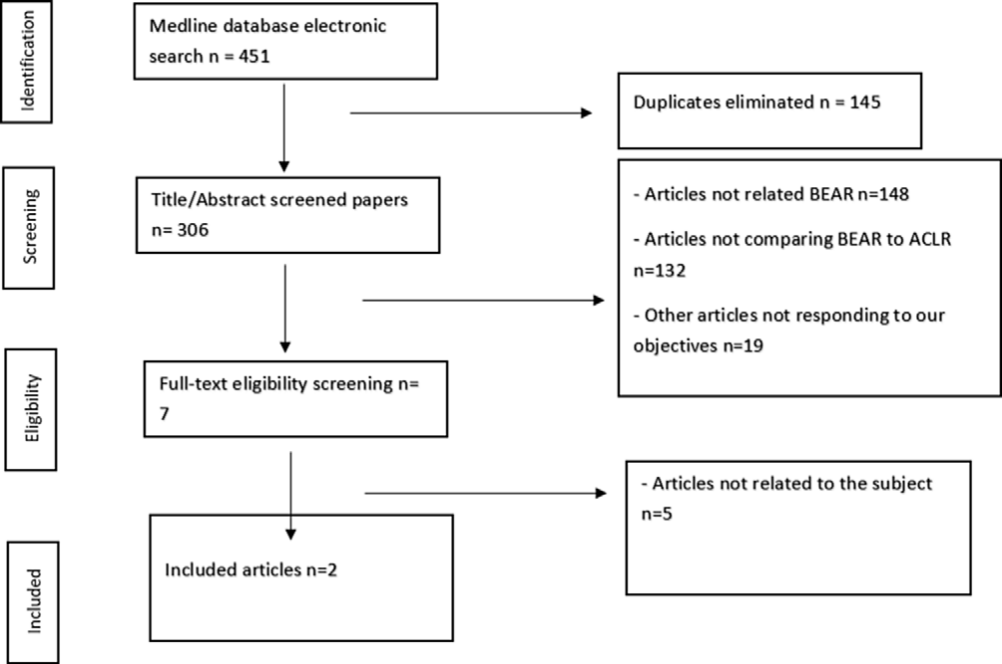
PRISMA flowchart for article selection process.

The PRISMA standards were followed in this investigation to compare BEAR and ACLR in the management of ACL tears. PubMed, Cochrane, and Google Scholar (pp. 1–20) searches were updated to November 2022 in search of qualified papers. Using Boolean Operators, a combination of the keywords “bridge” OR “BEAR” OR “Repair” AND “ACL” OR “Anterior Cruciate Ligament” was used. Reference lists from papers and online searches were also used to find literature. The data were extracted by one researcher (MD), and the article selection was verified by a different researcher (AG). The PRISMA flowchart provides a summary of the article selection process (Figure 1).

Inclusion criteria were (1) comparative studies: randomized controlled trials, prospective clinical trials, and retrospective studies; (2) clinical studies where patients were treated for ACL rupture; (3) clinical studies comparing patients who were treated using ACLR or BEAR technique. The studies with the following characteristics were excluded from this study: (1) case reports, narrative or systematic reviews, theoretical research, conference report, meta-analysis, cadaveric studies, expert comments, and economic analysis; (2) non-relevant outcomes or missing data (such as standard deviation).

### Data extraction

Two reviewers determined the eligibility of the studies independently. Extraction of the analyzed data was made from the included studies and it consisted of two parts. The first part consisted of the basic information containing the name of the authors, the title, the publication year, the journal, the volume, the issue, the pages, the study design, the sample size along with the size of each group of management (BEAR or ACLR), and the different types of bias suspected in each study. The second part consisted of the clinical outcomes at 2 years postoperatively which were the International Knee Documentation Committee (IKDC) score, Knee Injury and Osteoarthritis Outcome Score (KOOS), the side-to-side difference in Anteroposterior (AP) knee laxity, the forces of the hamstring, quadriceps, and hip abduction as well as hopping tests (single hop, triple hop, 6m hop, crossover hop). Any arising difference between the investigators was resolved by discussion.

### Risk of bias assessment

**Figure 2:**
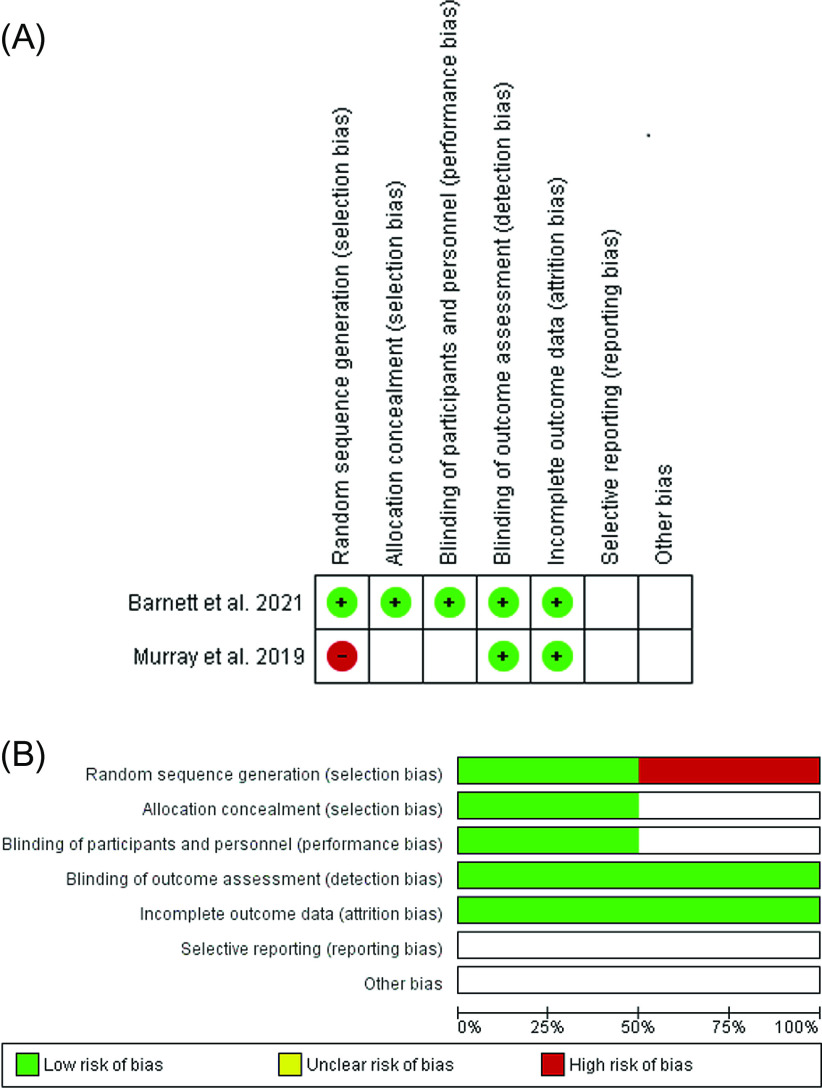
(A) Risk of bias item for each included study. (B) Risk of bias item presented as percentages across all included studies.

Two authors (MD and AG) used the Cochrane risk-of-bias tool to independently evaluate the risk of bias. Random sequence generation, allocation concealment, blinding of participants and study staff to the research procedure, blinding of outcome assessment, insufficient outcome data, and selective reporting were taken into account when determining whether a trial had a high, low, or unclear risk of bias (Figure 2A). A trial was assessed to have a high risk of bias if it had a high risk of bias for more than one key domain, whereas a trial would be considered to have a low risk of bias if it had a low risk of bias for every key domain. If neither of these conditions were met, the trials were deemed to have an unclear risk of bias.

### Statistical analysis

For statistical analysis, Review Manager 5.4 (The Cochrane Collaboration, 2020) software was used. Mean differences (MD) and 95% confidence intervals (CI) were utilized to analyze continuous data. *Q* tests and *I*^2^ statistics were used to evaluate heterogeneity if *p* ≤ 0.10 or *I*^2^ > 50% indicated considerable heterogeneity. High levels of variability in the variables were handled by the random-effect model. On the other hand, the fixed-effect model was chosen if *p* > 0.10 or *I*^2^ < 50%. Statistical significance is shown by *p* = 0.05.

## Results

### Characteristics of the included studies

Only two studies [14, 15] met the inclusion criteria with one prospective randomized comparative study, and one prospective non-randomized comparative study and were included in this meta-analysis. These involved 75 subjects in the BEAR group and 45 subjects in the ACLR group. The main characteristics of the included studies are summarized in Table 1. The results of the bias assessment for the included prospective studies are summarized in Figure 2B.

**Table 1:** Main characteristics of the included studies.

	Methods	Participants		Mean age (*SD*)		Measured outcomes	Follow-up time
		BEAR	ACLR	BEAR	ACLR		
Barnett et al. 2021 [14]	Randomized controlled trial	65	35	NA	NA	IKDC, KOOS, muscle strengths, hop test, Marx activity, KT1000 arthrometer for anteroposterior knee laxity, ROM flexion-extension	24 months
Murray et al. 2019 [15]	Prospective non-randomized comparison	10	10	24.1 (4.9)	24.6 (5.5)	IKDC, KOOS, side-to-side difference in anteroposterior (AP) knee laxity, muscle strengths, hop test	24 months

### Functional outcomes

#### IKDC score

**Figure 3:**
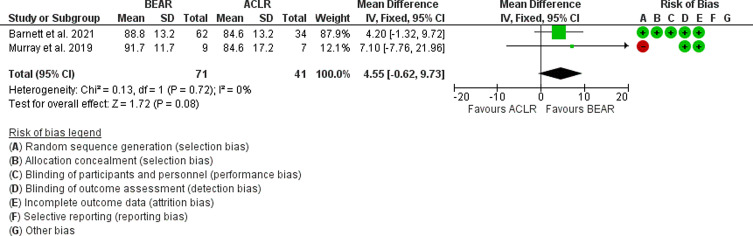
Forest plot showing the IKDC scores 2 years postoperatively in ACLR and BEAR.

The results showed no statistical difference between BEAR and ACLR. (*p* = 0.08, Mean difference = 4.55; 95% CI: −0.62 to 9.73, Figure 3).

#### KOOS score

**Figure 4:**
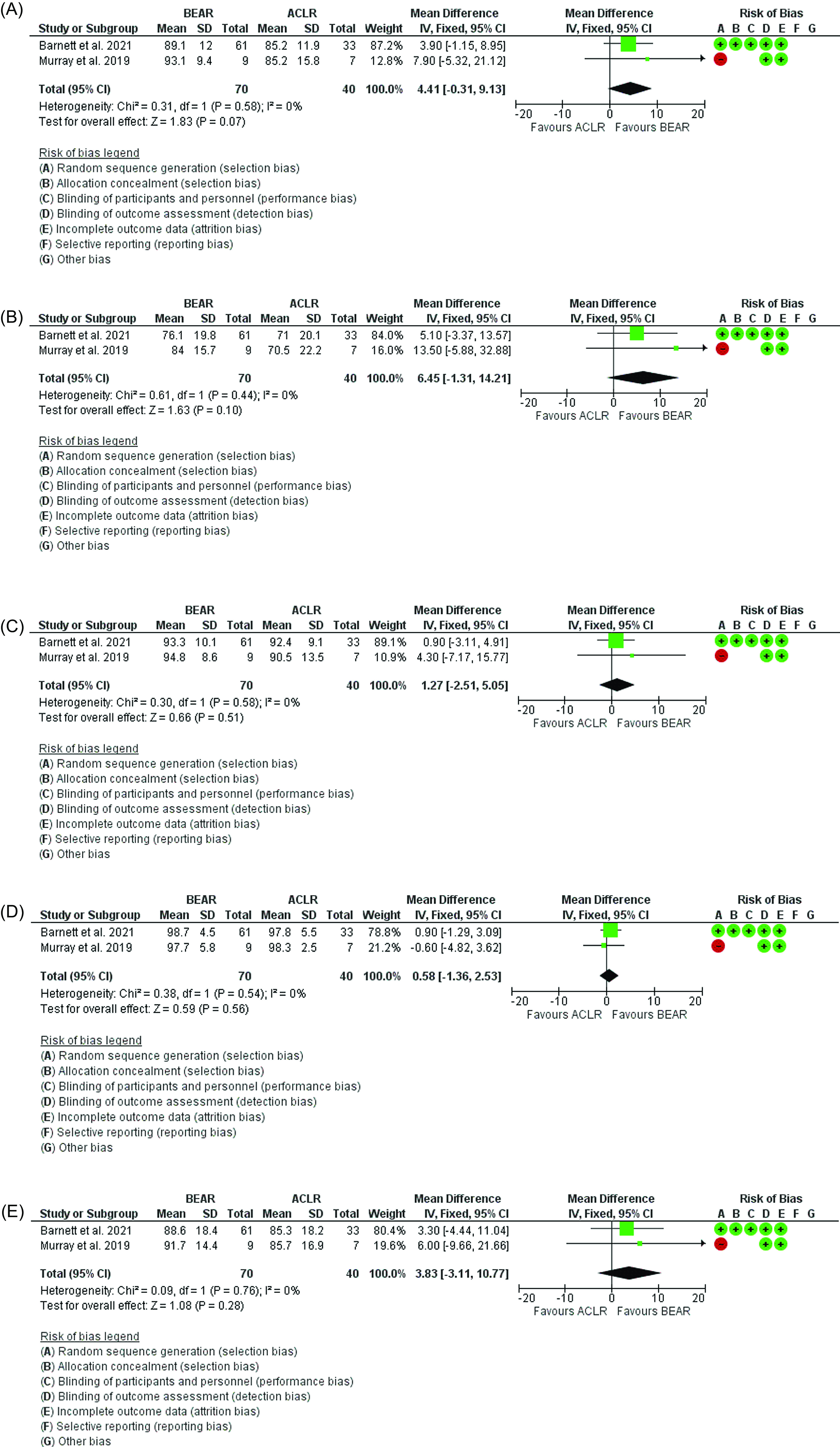
(A) Forest plot showing the KOOS scores 2 years postoperatively (symptoms section) in ACLR and BEAR. (B) Forest plot showing the KOOS scores 2 years postoperatively (knee-related quality of life section) in ACLR and BEAR. (C) Forest plot showing the KOOS scores 2 years postoperatively (pain section) in ACLR and BEAR. (D) Forest plot showing the KOOS scores 2 years postoperatively (activities of daily life section) in ACLR and BEAR. (E) Forest plot showing the KOOS scores 2 years postoperatively (sports and recreation section) in ACLR and BEAR.

The results showed that no statistical difference between BEAR and ACLR in regard to the symptoms (*p* = 0.07, Mean difference = 4.41; 95% CI: −0.31 to 9.13, Figure 4A), knee-related quality of life section (*p* = 0.1, Mean difference = 6.45; 95% CI: −1.31 to 14.21, Figure 4B), pain (*p* = 0.51, Mean difference = 1.27; 95% CI: −2.51 to 5.05, Figure 4C), activities of daily life (*p* = 0.56, Mean difference = 0.58; 95% CI: −1.36 to 2.53, Figure 4D), and sports and recreation (*p* = 0.28, Mean difference = 3.83; 95% CI: −3.11 to 10.77, Figure 4E).

### AP knee laxity

**Figure 5:**
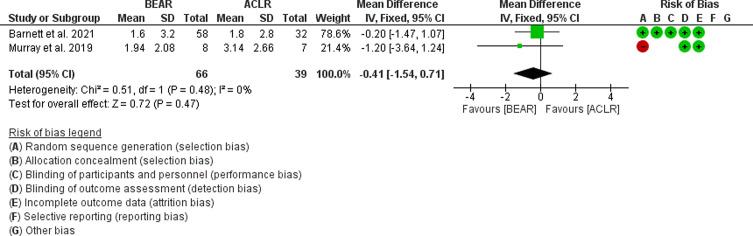
Forest plot showing the side-to-side difference in AP knee laxity 2 years postoperatively in ACLR and BEAR.

The results showed that there was no statistical difference in AP knee laxity 2 years postoperatively (*p* = 0.47, mean difference = −0.41; 95% CI: −1.54 to 0.71, Figure 5).

### Muscle strength

**Figure 6:**
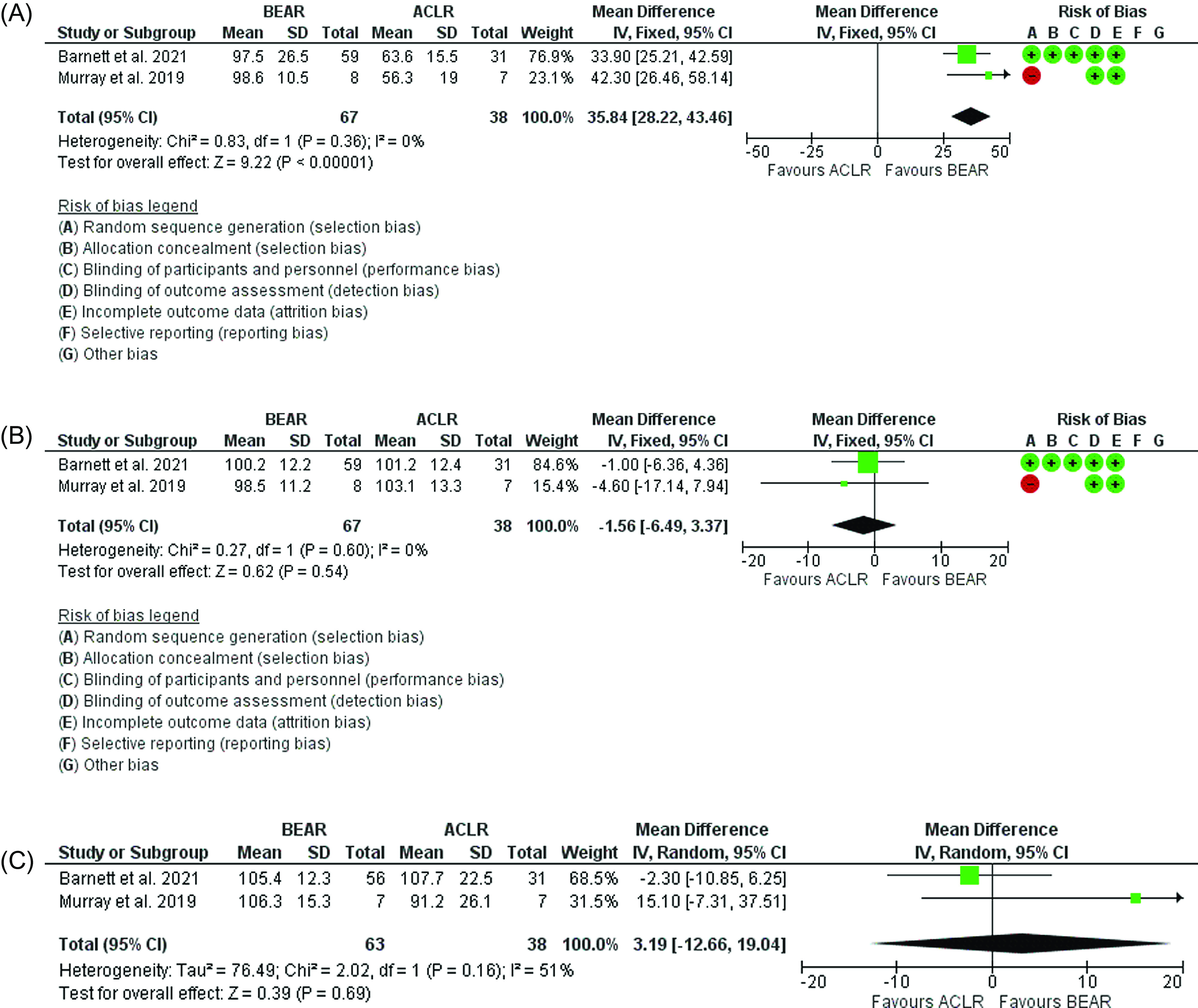
(A) Forest plot showing the hamstrings strength 2 years postoperatively in ACLR and BEAR. (B) Forest plot showing the quadriceps strength 2 years postoperatively in ACLR and BEAR. (C) Forest plot showing the hip abductors strengths 2 years postoperatively in ACLR and BEAR.

The results showed that when compared to ACLR, BEAR had a significantly better hamstring strength 2 years postoperatively. (*p* < 0.00001, mean difference = 35.84; 95% CI: 28.22 to 43.46, Figure 6A). However, when comparing quadriceps (*p* = 0.54, mean difference = −1.56; 95% CI: −6.49 to 3.37, Figure 6B) and abductors strengths (*p* = 0.69, mean difference = 3.19; 95% CI: −12.66 to 19.04, Figure 6C) there was no difference between the ACLR and BEAR technique.

### Hop tests

**Figure 7:**
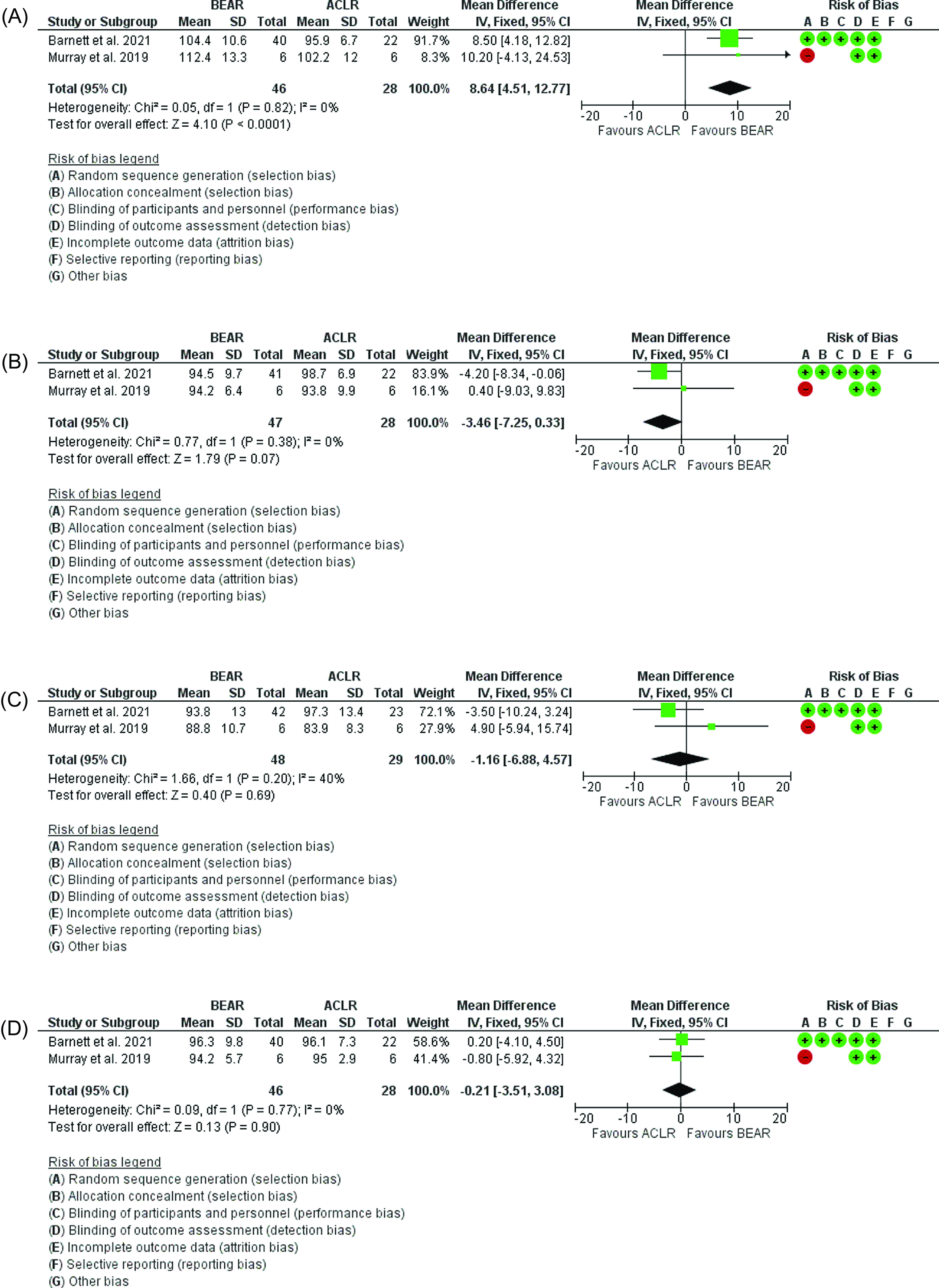
(A) Forest plot showing the 6m hop 2 years postoperatively in ACLR and BEAR. (B) Forest plot showing the triple hop 2 years postoperatively in ACLR and BEAR. (C) Forest plot showing the single hop 2 years postoperatively in ACLR and BEAR. (D) Forest plot showing the crossover hop 2 years postoperatively in ACLR and BEAR.

The results showed that when compared to ACLR, the BEAR group had a significantly better 6m distance hop 2 years postoperatively (*p <* 0.0001, mean difference = 8.64; 95% CI: 4.51 to 12.77, Figure 7A). However, when comparing the triple hop, the results showed that no statistical difference (*p* = 0.07, mean difference = −3.46; 95% CI: −7.25 to 0.33, Figure 7B) as well as in the single hop test (*p* = 0.69, mean difference = −1.16; 95% CI: −6.88 to 4.57, Figure 7C) and crossover hop test (*p* = 0.9, mean difference = −0.21; 95% CI: −3.51 to 3.08, Figure 7D).

## Discussion

ACL tears are a common injury seen in athletes. For a long time, ACLR was the standard of care when faced with such an injury [16]. Nowadays, primary repair techniques are emerging such as the BEAR technique and the results are appearing to be comparable. However, there is still no strict consensus on which technique is superior. Our meta-analysis aims first to compare ACLR to the BEAR technique for the management of ACL tears knowing that both showed similar outcomes.

A study performed by Barnett et al. [16] revealed no difference in postoperative opioid use when comparing ACLR to BEAR. What was indicated to influence the consumption of painkillers is the body mass index as well as the amount of pain pre-operatively [16]. Both of these factors are not surgery related, and this supports the results seen in our study concerning postoperative pain. One of the main reasons why no difference in postoperative pain between ACLR and BEAR was observed is the fact that this tissue scaffold is tolerated by the articular synovium of the knee resulting in decreased infections, serious inflammatory reactions, and secondary surgery for scaffold removal [13]. Moreover, there was no difference in the failure rate between both groups [17]. Furthermore, when comparing magnetic resonance imaging 3 months postoperatively between ACLR and BEAR groups, there was no significant difference in the knee synovium [13]. Reasons behind the decreased inflammatory reaction around this BEAR scaffold which is still considered as a foreign body is the fact that this implant is not crosslinked allowing for cells to permeate through this bridge 1 week postoperatively [18], the implant contains very little xenogeneic DNA [19, 20], it was treated by an enzyme that removes the 3’ and 5’ ends of the collagen leaving only the center of the molecule [13], and the scaffold is made to be hydrophilic making the absorption of the patient’s blood easier [13].

Our study revealed similar IKDC scores as well as KOOS scores in both techniques. A randomized controlled trial by Barnett et al. [14] showed that earlier in the postoperative period, the difference in both the IKDC and KOOS scores was higher in favor of the BEAR group and this difference diminished over time. This can be explained by an earlier symptom resolution and return of function in the BEAR group. Reasons behind this earlier resolution can be the absence of the donor site morbidity in the BEAR procedure leading to reduced postoperative symptoms, an earlier remission of knee issues, and increased patient satisfaction, particularly in the first several months following surgery [14].

Hamstring strength was significantly better in the BEAR group with no difference in the strength of hip abductors or quadriceps muscles. This difference in hamstring strength was seen early in the postoperative period and maintained with no reduction in this difference until 2 years postoperatively as opposed to the KOOS and IKDC scores [14]. This outcome is very important since hamstring tendons are acknowledged to dynamically enhance the function of the ACL, and hamstring weakness persisting over time has been linked to future knee injury [21, 22]. The ACLR group may be at a greater risk of reinjury as a result of the observed variations in hamstring strength [14]. The reason behind this finding may be associated with graft harvesting from the hamstrings in ACLR [14]. Persistent loss of hamstring strength in ACLR is not uncommon when the graft is harvested from these muscles [23–25]. To mediate this loss of strength, the graft can be taken from the patellar tendon, which is why a study comparing BEAR to ACLR using patellar tendon autograft could shed light on this outcome.

With regard to the hopping tests, the BEAR group had a better 6m distance hop result however, the rest of the results were comparable. This difference in these outcomes is not seen to be detrimentally affected by the difference in hamstring strength [17]. The clinical value and impact of these outcomes are yet to be fully understood. In addition, Murray et al. showed a rate of 14% of ACL re-rupture in the BEAR group compared to a 6% rate in the ACLR group, however, the difference was not statistically significant [17]. In fact, our results showed no difference in AP knee laxity between ACLR and BEAR.

A study by Sanborn et al. [26] compared the ACL Return to Sports after Injury (ACL-RSI) score in both the BEAR and ACLR techniques. The ACL-RSI cut-off score is set to be 65 as per Webster et al. [27], 6 months postoperatively, the BEAR group attained this score while the ACLR group did not, however at 12 months postoperatively both groups preserved the same psychological readiness making this delay in psychological readiness a temporary and not a permanent issue. This can be explained by the fact that patients may have been more confident earlier in their postoperative course, due to the lack of donor-site morbidity from graft harvest in the BEAR group and, presumably, faster muscle recovery. Our findings showed better hamstring strengths in the BEAR group and a correlation between better psychological readiness scores and higher hamstring strength at 6 months was noted by Sanborn et al. [26], which supports the notion of a mind-body connection during rehabilitation. Other factors that had a major influence on this score at 6 months are the participation in level 1 sports before the injury, and the postoperative IKDC score [26]. This finding suggests that the patient’s perceived knee function matches psychological readiness and that athletes may also have stronger athletic identities, which motivates them to adhere more strictly to recovery goals [28, 29].

### Strengths and limitations

This study presents some limitations, mainly the number of included studies is limited, and the fact that the data used for analysis were pooled and individual patients’ data were unavailable which could limit further comprehensive analyses. Moreover, most of the patients in the ACLR group had a reconstruction of the ACL using the hamstrings tendon and not the patellar tendon which is stronger. However, we only chose comparative studies to be included, thereby reducing the risk of operative and matching bias and the selection process was meticulous and discerning, making the study less heterogeneous and decreasing the risk of bias. Furthermore, this study is the first study comparing BEAR to ACLR in the management of ACL tears.

## Conclusion

This is the first meta-analysis comparing the BEAR and the ACLR technique in the management of ACL tears. Compared to the ACLR technique, the BEAR surgery showed no differences in muscle strength (quadriceps and hip abductors) knee joint laxity, and postoperative knee scores. However, it showed better hamstring strength. Earlier resolutions of symptoms and return to activities were also seen in the BEAR group. These results prove that this primary repair technique is a reliable and efficacious technique for the treatment of ACL ruptures, however, further randomized clinical studies will be needed to compare both of these techniques.

## Data Availability

Available upon request from the corresponding author.
